# Placental mesenchymal stem cell–derived exosomes treat endometrial injury in a rat model of intrauterine adhesions

**DOI:** 10.1007/s00438-025-02241-x

**Published:** 2025-03-25

**Authors:** Lin Liang, Huidong Liu, Shaowei Wang

**Affiliations:** 1 Department of Gynecology and Obstetrics, Beijing Hospital, National Center of Gerontology, Institute of Geriatric Medicine, Chinese Academy of Medical Sciences, No. 1, Dahuaroad, Dongdan, Beijing, 100730 P.R. China; 2 Peking Union Medical College, Chinese Academy of Medical Sciences, Graduate School of Peking Union Medical College, Dong Dan Santiao, Beijing, 100730 P.R. China

**Keywords:** Placental mesenchymal stem cell, Exosomes, Intrauterine adhesion, NF-κB signaling, MyD88, Immunity

## Abstract

Intrauterine adhesion (IUA) refer to persistent inflammation and fibrosis due to damaged or infected endometrium and eventually lead to dysfunction. This study aimed to explore the therapeutic effects of exosomes (Exos) derived from placental mesenchymal stem cells (PMSCs) on endometrial repair in a rat model of IUA and to elucidate the underlying molecular mechanisms. PMSCs were characterized using flow cytometry and differentiation assays (osteogenic, adipogenic, and chondrogenic). Exos were isolated via ultracentrifugation and validated through transmission electron microscopy, nanoparticle tracking analysis and Western blot. An IUA model was established via electrocoagulation, and endometrial repair was assessed using hematoxylin-eosin (HE) and Masson staining. RNA sequencing, differential expression analysis and protein-protein interaction (PPI) network construction were employed to investigate the molecular mechanisms of PMSC Exos mediated repair. The role of miR-143 in targeting MyD88 and modulating the NF-κB signaling pathway was confirmed using Dual-Luciferase Reporter Assay and qRT-PCR. PMSC Exos significantly improved endometrial thickness, increased glandular number and reduced fibrosis in the IUA model. RNA sequencing and differential expression analysis screened 3980 differentially expressed genes (DEGs) common to the IUA vs normal groups and Exo vs IUA groups. Enrichment analysis revealed significant involvement of immune system processes, natural killer cell-mediated cytotoxicity and NF-κB signaling. PMSC Exos delivered miR-143, which targeted MyD88, thereby regulating the NF-κB pathway. PMSC Exos effectively repaired endometrial damage in the IUA model by modulating the NF-κB signaling pathway through miR-143 delivery. These findings suggest that PMSC Exos hold promise as a novel therapeutic strategy for IUA, offering insights into the molecular mechanisms underlying endometrial repair.

**Supplementary Information:**

The online version contains supplementary material available at 10.1007/s00438-025-02241-x.

## Background

Intrauterine adhesion (IUA), also known as Asherman’s syndrome, refers to a pathological condition characterized by abnormal adhesion and scar formation in the uterine endometrium. This condition is typically caused by uterine surgery, endometrial inflammation, postpartum infection, induced abortion, or other factors that result in uterine endometrial damage (Leung et al. 2021). The formation of IUA can have a profound impact on women’s reproductive health, including menstrual abnormalities, infertility, recurrent miscarriages, and premature births (Kelleher et al. 2018). Approximately 21.2% of patients with IUA experience secondary infertility (Koskas et al. 2010), and 30% of women experience spontaneous abortion in late pregnancy due to IUA (Liu et al. 2022). Despite the availability of conventional treatment methods, the recurrence rate for mild-to-moderate patients with IUA remains 30%, and severe cases have a recurrence rate of as high as 62.5% (Chen et al. 2017). Therefore, developing effective strategies for endometrial regeneration remains a critical challenge in IUA management.

In recent years, various types of stem cells, particularly mesenchymal stem cells (MSCs), have garnered significant interest in the field of reproductive medicine because they promote multilineage differentiation and tissue repair and regeneration, improve reproductive organ function, and have low immunogenicity and wide availability (Gao et al. 2022). For example, bone marrow stromal cells (BMSCs) can effectively reconstruct damaged endometrium and improve reproductive outcomes by reducing the degree of endometrial fibrosis (Gao et al. 2022). Umbilical cord marrow stromal cells (UCMSCs) induction markedly increased the number of endometrial glands and reduced fibrotic area in rats with induced IUA (Liu et al. 2020). Human menstrual blood-derived MSCs were expanded in vitro and transplanted into the uteruses of women with intractable IUA. In another study, five of 12 patients achieved clinical pregnancy, resulting in a pregnancy rate of 41.7% (Ma et al. 2020). MSCs derived from amniotic membrane promoted endometrial regeneration in a rat model of IUA (Mao et al. 2023). Although MSC therapy has demonstrated promising therapeutic efficacy, limitations, such as low engraftment rates, loss of stemness, inconvenience of transportation and storage, and uncontrolled differentiation, still restrict the clinical application of MSCs (Xin et al. 2020). Current research indicates that MSCs play a crucial role in tissue repair, and 80% of their effectiveness is attributed to their paracrine factors, such as chemokines, growth factors, and cytokines, which are secreted into the surrounding environment (Costa et al. 2021).

Exosomes (Exos) are small membrane-bound vesicles released by cells, with diameters ranging from 50 nm to 150 nm (Qiu et al. 2019). They contain a variety of paracrine factors produced by cells. The concept of MSC-derived Exo therapy is to utilize the secreted factors to promote tissue repair and regeneration. Compared with MSCs therapy, extracellular vesicle therapy is more feasible and promising because it is a cell-free treatment approach that is easier to manage and offsets certain limitations and risks associated with traditional stem cell therapy while retaining the benefits of cell-based treatments (Lee et al. 2021). Exos derived from the MSCs of the bone marrow can reverse epithelial–mesenchymal transition (EMT) through the TGF-β1/Smad pathway, facilitating the repair of damaged endometrium (Yao et al. 2019). Exos derived from adipose-derived MSCs restored endometrial function in a rat model of IUA (Zhao et al. 2020), and Exos generated by UCMSCs play a crucial role in reversing IUA formation. This effect is facilitated by the miR-145-5p/zeb2 axis, which reverses endometrial fibrosis (Li et al. 2023). However, although numerous studies have focused on the treatment of IUA with MSC Exos, no research regarding the application of Placental mesenchymal stem cells (PMSCs) secreted Exos to IUA treatment has been conducted. Compared with other MSCs, PMSCs have a richer source, lower immunogenicity, higher proliferative capacities, and stronger multipotent differentiation potential (Maraldi and Russo 2022). The treatment approach involving the secretion of Exos by PMSCs holds promise as a novel and feasible cellular-free therapy strategy for endometrial regeneration (Liu et al. 2019).

In this study, we investigated for the first time the therapeutic effect of PMSC Exos on IUA and conducted transcriptome sequencing to explore the underlying mechanisms. Through a series of bioinformatics analyses, we investigated the pathways potentially involved in the formation and treatment process of IUA . Additionally, we found that PMSC Exos may exert their therapeutic effects by delivering their enriched miR-143 to target MyD88, thereby regulating the NF-κB signaling pathway.

## Methods

### PMSC identification

The PMSCs of the third passage were identified. When the cells reached 70%–80% confluency, the complete medium was replaced with an adipogenic induction medium for 3 weeks. Then, Oil Red O staining was performed. When the cells reached 60%– 70% confluency, the complete medium was replaced with an osteogenic induction medium for 3 weeks. Then, Alizarin Red staining was performed. When the cells reached 70%–80% fusion, the complete culture medium was replaced. When the cell fusion reached 70%–80%, the complete culture medium was replaced with a chondroblast culture medium for 4 weeks. Moderate changes were made twice a week, and cartilage formation was assessed every 2–3 days. Cartilage differentiation was then assessed with Alcian blue staining. In addition, flow cytometry was used to detect the expression of PMSCs surface markers CD73, CD90, and CD105.

### Exosome extraction and identification

Exos were extracted through ultrafast centrifugation. When the cell confluence reached 80%, the serum-free medium was replaced. After 48–72 h of culture, the cell culture supernatant was collected, and dead cells and large extracellular vesicles were removed through centrifugation at 4 °C for 3000 g of the supernatant for 30 min. The cell fragments were then removed through centrifugation at 4 °C for 10,000 g for 30 min, and the supernatant was filtered with a 0.22 μm filter into a 50 ml centrifugation tube. Approximately 100,000 g of the supernatant was centrifuged at 4 ℃ for 90 min, and the supernatant was discarded to collect precipitation. The Exos were resuspended with PBS. BCA protein quantification was used to detect Exos protein concentration. The morphology of Exos was observed under a transmission electron microscope, and the peak particle size of Exos was revealed through nanoparticle tracking analysis. Western blot (WB) was performed for the identification of the Exo-specific marker CD63 (1:1000; Proteintech, America) and HSP70 (1:1000; Proteintech, America).

### Animals

Female rats aged 10–12 weeks were randomly divided into control, model, and Exo treatment groups. An IUA model was established by using a previously described electrocoagulation damage method (Liu et al. 2019). After the administration of anesthesia with isoflurane through inhalation, a laparotomy was performed, and the uteruses were exposed. A small incision was made on the left side, an electrocoagulation needle was inserted, and the tip was moved from the corner of the uterus to the cervix. A power of 20 W was used to deliver the current. After injury, a syringe was used to inject different therapeutic fluids directly into the uterine cavity through the previous incision. The Exo treatment group was treated with 500 µL of Exos (1 mg/mL), and the IUA group, with 300 µL of normal saline. The right uterus was not treated for comparison and reduction of experimental error and interference. Suture the incision after treatment. After 20 days of treatment, the rats were killed, and uterine tissues were collected for subsequent experiments. All animals were obtained from Beijing Vital River Laboratory Animal Technology Co., Ltd. (Beijing, China). Animal experiments were performed in compliance with the guidelines of Beijing Laboratory Animal Management Office (MDSW-2023-048C) .

### Hematoxylin–eosin (HE) and masson staining

Uterus tissues from each group were fixed in 4% neutral buffered formalin, dehydrated, and embedded in paraffin. Serial sections of 4 μm were sliced and stained with hematoxylin–eosin (HE) and Masson staining. Four 20× magnification regions were selected for each HE-stained section, and the number of glands in each field of view was calculated and averaged. Four 10× magnification areas were selected for each HE-stained section, and the endometrial thickness in each field of view was calculated and averaged. Four 40× magnification areas were selected for each Masson-stained section, and the fibrosis area ratio was calculated as follows: total endometrial fibrosis area per field of view/total endometrial matrix and gland area. The rate was automatically averaged by using ImageJ (Rawak Software, Inc., Germany).

### Microarray and mRNA bioinformatics analysis

The analysis of miRNA and mRNA encoding proteins utilized Illumina Novaseq 6000 (LC Biotechnology Co., Ltd., Hangzhou, China) for dual-end sequencing according to standard protocols. The sequencing mode employed was PE150. GeneSpring v13.0 was used for miRNA + mRNA array data analysis including data summarization, normalization, and quality control. Principal component analysis (PCA) and Pearson correlation analysis were used in analyzing the samples, and |log2fc ≥1 and p <0.05 were used as threshold values in the selection of differentially expressed genes (DEGs) for the comparison of IUA and normal groups and of Exo and IUA groups. Venn diagrams were used to show the shared DEGs between the IUA vs normal group and the Exo vs IUA group. We performed GO/KEGG and GSEA enrichment analyses on the DEGs to determine potential underlying mechanisms for this process. Subsequently, the DEGs were analyzed by using the STRING plugin in Cytoscape v3.9.1, and the String Enrichment plugin was applied to visualize the protein–protein interaction (PPI) network of key pathways. Venn diagrams were used to display the miRNAs shared among the IUA vs normal group, the Exo vs IUA group and the top 30 miRNAs in exosomes. The target genes of these miRNAs were screened using the TargetScan (https://www.targetscan.org/vert_80/) and StarBase databases(https://rnasysu.com/encori/).

### Quantitative real-time reverse transcription polymerase chain reaction (RT-PCR)

To verify the expression of key miRNAs, we performed RT-PCR. Total RNA was extracted from the cells by using an miRNeasy mini kit (Qiagen, Germany) according to the manufacturer’s protocol. The reverse transcription and polymerase chain reaction (PCR) primers for miRNAs and U6 were obtained from RiBoBio (Guangzhou, China) and are listed in Table 1. A cDNA library was constructed by using a PrimeScript RT reagent kit (Takara, Japan). For the quantification of mature miRNA, cDNA was generated with specific stem-loop universal primers. qRT-PCR for miRNA was performed by using SYBR Premix Ex Taq II (Takara) and was measured by using an ABI 7500 sequence detection system. U6 was used as the internal control. The relative amount of miRNA was calculated using the 2^–∆∆Ct^ method.

**Table 1:** List of qRT-PCR primers used in this study

Gene name	Primer name	Sequence (5′ → 3′)
miR-143-3p	miR-143-3p-stem-loop	CTCAACTGGTGTCGTGGAGTCGGCAATTCAGTTGAGTGTAGCTC
	miR-143-3p-F	ACACTCCAGCTGGGTGAGATGAAGCACTGTA
	miR-143-3p-R	CTCAACTGGTGTCGTGGA
U6	U6-F	CTCGCTTCGGCAGCACA
	U6-R	AACGCTTCACGAATTTGCGT
MyD88	MyD88-F	TTGCCAGCGAGCTAATTGAG
	MyD88-R	ACAGGCTGAGTGCAAACTTG
GAPDH	GAPDH-F	TCTCCCTCACAATTTCCATCCC
	GAPDH-R	TTTTTGTGGGTGCAGCGAAC

### Dual-luciferase reporter assay

To determine if MyD88 is the direct targets of miR-143, the luciferase reporter test was carried out on 293T cells. The 3’-untranslated region (3’-UTR) of MyD88 containing the miR-143 binding site was cloned to produce a pMIR-MyD88-WT vector, whereas the pMIR-MyD88-Mut vector was constructed using 3’-UTR that did not contain miR-143. Subsequently, the pMIR-MyD88-WT vector or pMIR-MyD88-Mut vector was cotransfected into cells with either a miRNA mimic or NC. The cells were inoculated in 24-well culture plates. After 48 h, the luciferase activity was determined (Vazyme, Najing, China).

### Statistical analysis

R (4.2.1 version) and GraphPad Prism 9 were utilized for statistical analysis. The Mann–Whitney U test and unpaired two-tailed Student’s t-tests were employed to compare the groups. The Pearson method was used to assess correlation among samples, and the Spearman method was employed to evaluate correlation among mRNAs. Statistical significance was defined as p <0.05.

## Results

### Identification of PMSCs and its exosomes

**Fig. 1:**
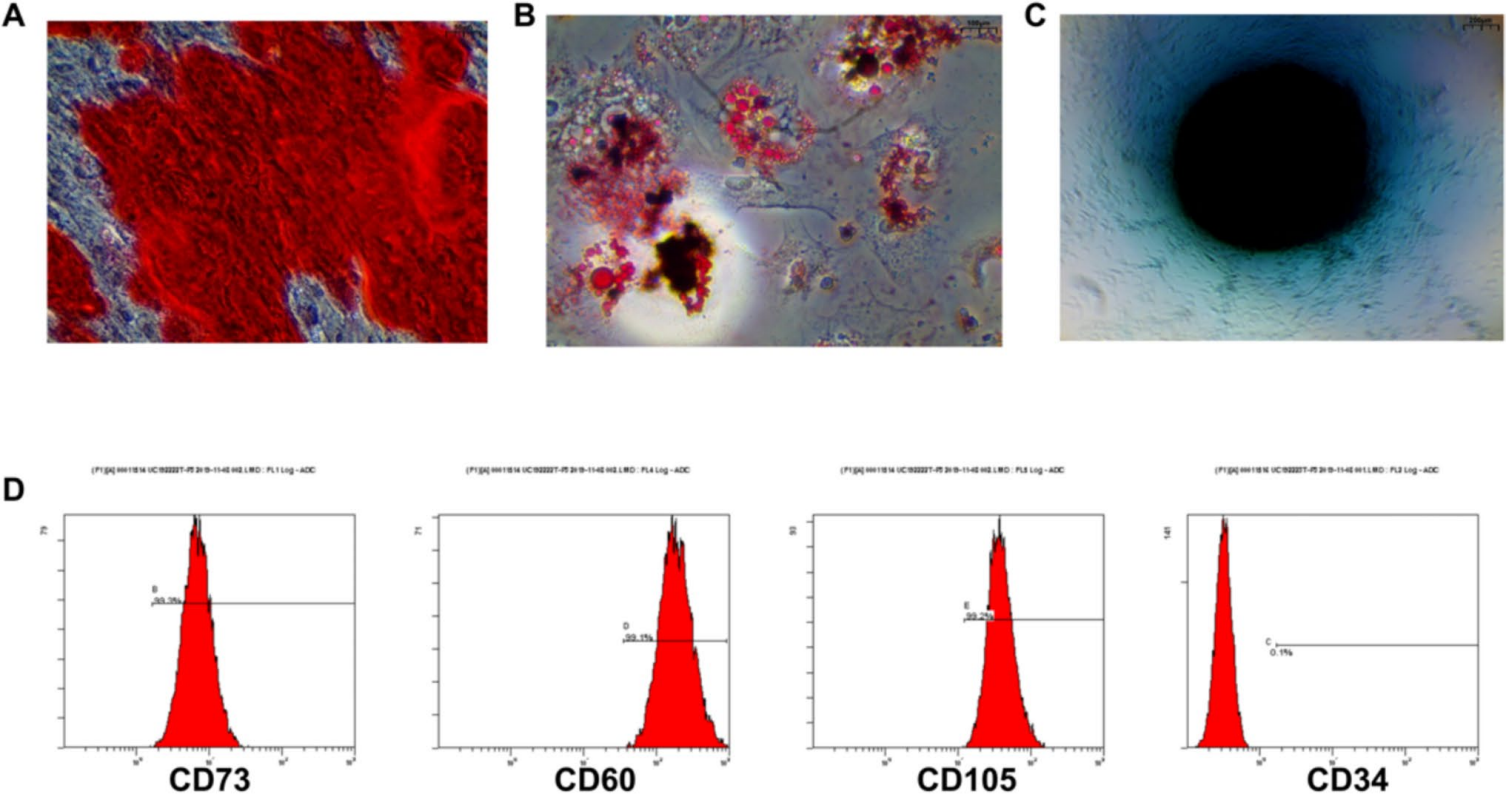
Identification of PMSCs **A**: Osteogenic induction of PMSCs. **B**: Adipogenic induction of PMSCs. **C**: Chondrogenic induction of PMSCs. **D**: Detection of CD90, CD73, CD105 and CD34 by flow cytometry

**Fig. 2:**
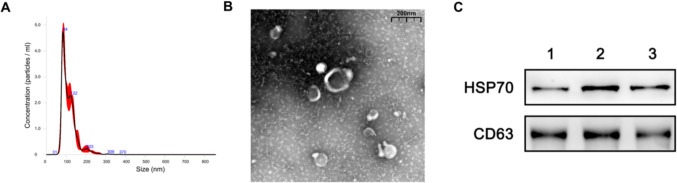
Identification of PMSC Exos **A, B**: Exosomes derived from PMSCs were observed by TEM and NTA. **C**: WB was used to detect the specific surface proteins of the exosomes CD63 and HSP70

After osteogenic differentiation induction of PMSCs, calcium nodules were formed via Alizarin Red staining (Figure 1a). After PMSCs were induced to differentiate into adipogenesis, Oil Red O staining showed obvious lipid droplets in the cells (Figure 1b). After we induced the chondrogenic differentiation of PMSCs, blue-stained acid proteoglycans formed, as indicated by Alcian blue staining results (Figure 1c). Flow cytometry results showed that the PMSCs were positive for the markers of mesenchymal stromal stem cells, namely, CD90, CD73, and CD105, and negative for hematopoietic markers, namely, CD34 (Figure 1d). Transmission electron microscopy (TEM) showed that the Exos were round or appeared as oval membrane vesicles, and the peak particle size of Exos was approximately 84 nm according to nanoparticle tracking analysis (NTA; Figures 2a,b). The Exo markers CD63 and HSP70 were well expressed according to WB results (Figure 2c).

### PMSC Exos improved endometrial injury in the rat models

**Fig. 3:**
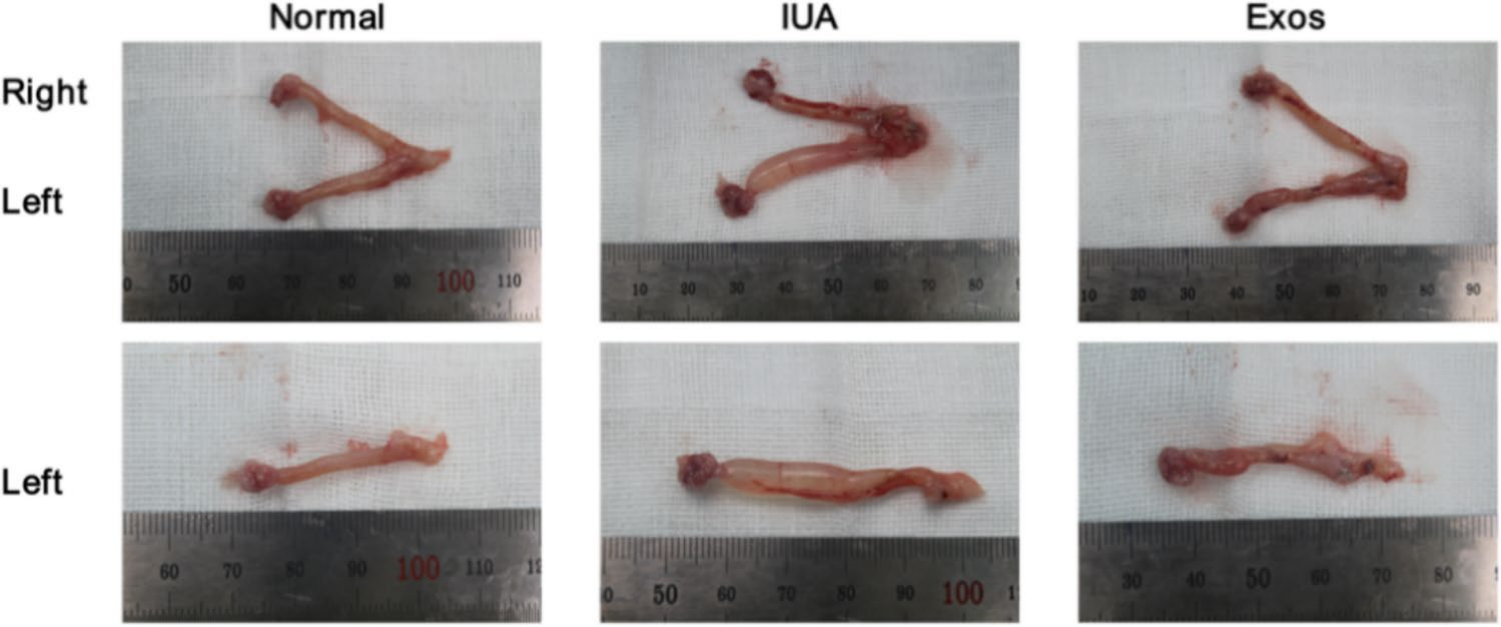
Uterus specimen

**Fig. 4:**
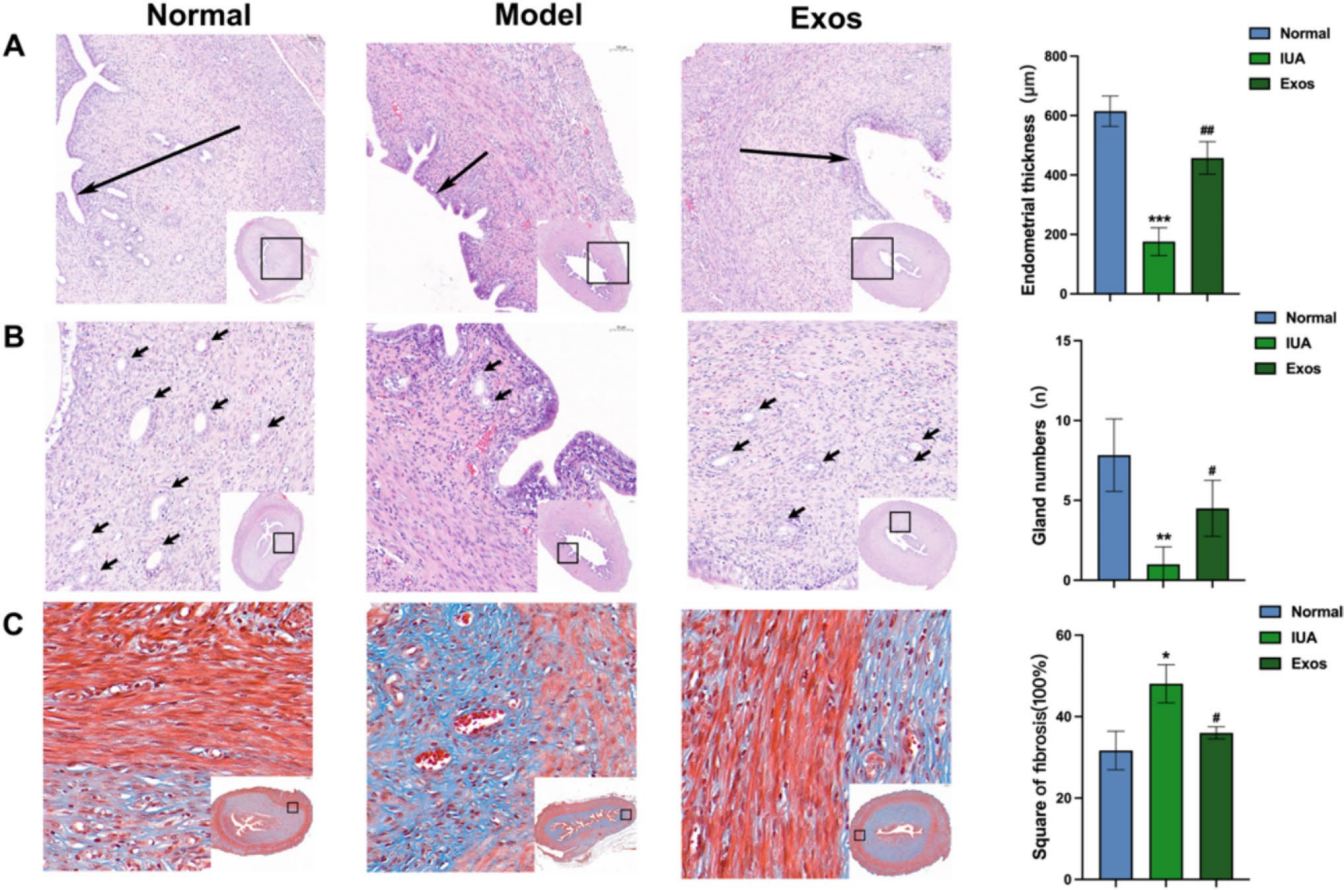
PMSC Exos improved endometrial injury in the rat models **A**: The HE staining of rat uterine tissue (10×) and statistical analysis of endometrial thickness. **B**: The HE staining of rat uterine tissue (20×) and statistical analysis of number of glands. **C**: The Masson staining of rat uterine tissue (40×) and statistical analysis of endometrial fibrosis (*compared with the normal group; # compared with the IUA group)

Animal experiments were divided into normal, IUA, and Exo groups. After 14 days of treatment, uterine tissues were extracted from the rats. As shown in Figure 3, the left side of the uterus showed significant edema and was structurally deformed in the IUA group compared with the normal group. By contrast, the Exos treatment group only showed fluid accumulation at the end of the uterus and mild congestion and edema. These results showed that PMSC Exos alleviated edema and congestion in the rat IUA model and reduced the accumulation of fluids. Three groups of uterine tissues were selected for HE staining for the detection of changes in endometrial thickness and number of glands. Endometrial thickness was calculated at 10× magnification. Endometrial thickness was significantly reduced in the IUA group compared with that in the normal group, and the endometrial thick floor significantly improved after Exo treatment (Figure 4a). The glands were counted as 20× magnification. The average number of glands in the normal group was 7.8, whereas that in the IUA group was only 1. The number of glands in the IUA model was greatly reduced, and the average number of glands increased to 4.5 after Exo treatment (Figure 4b). Changes in endometrial fibrosis were then assessed through Masson staining. The fibrosis level was significantly higher in the IUA group than in the normal group. The level of fibrosis was reduced in the Exo group compared with the model group (Figure 4c). These results indicated that Exos can restore the morphology of a damaged endometrium, increase endometrial thickness and number of glands, and improve fibrosis.

### Analysis and identification of the DEGs

**Fig. 5:**
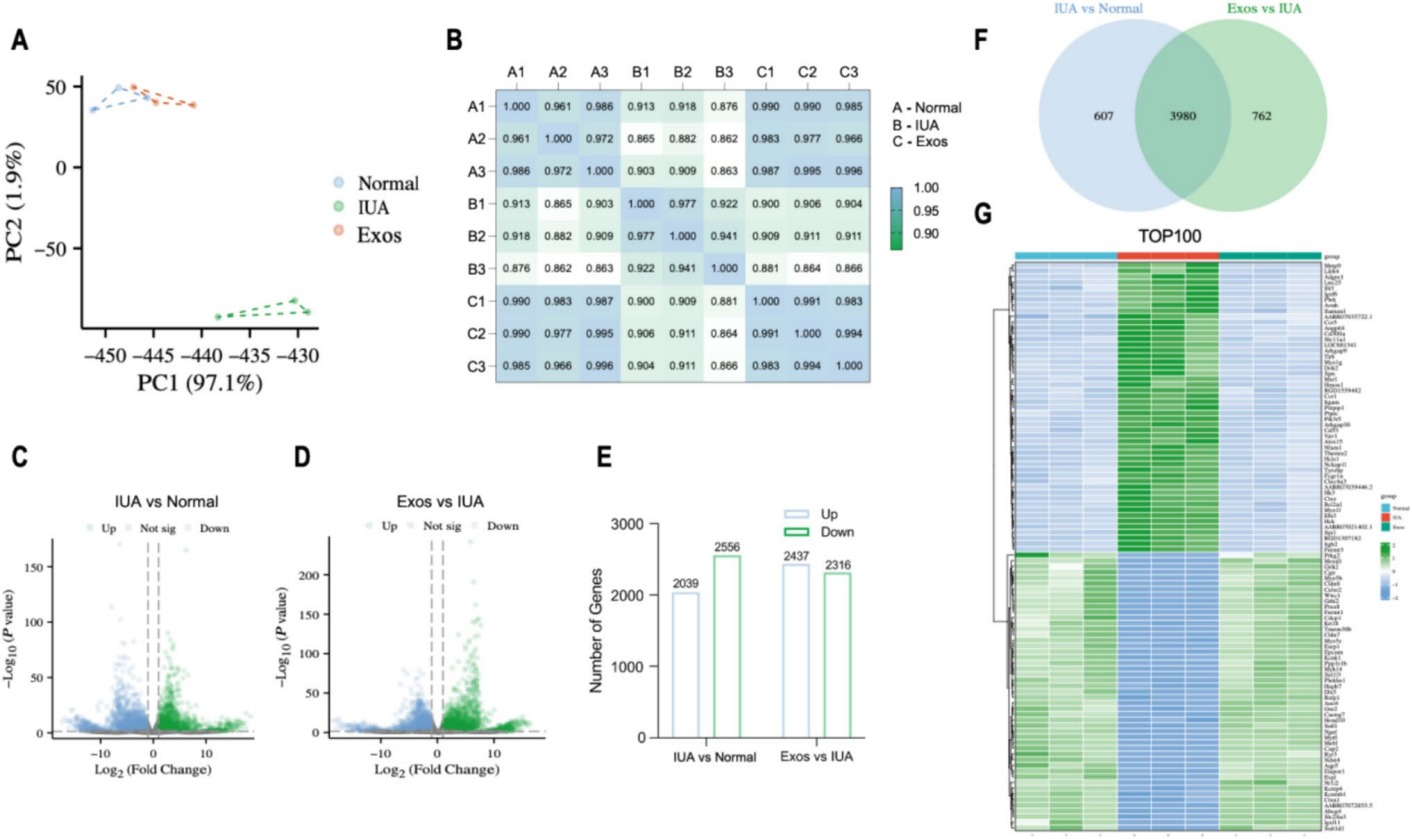
Analysis and identification of differentially expressed genes **A**: PCA of the distinct mRNAs based on the microarray assay. **B**: Pearson correlation analysis of different microarray samples. **C**: A volcano plot of the distinct mRNAs based on IUA vs. normal groups. **D**: A volcano plot of the distinct mRNAs based on Exos vs IUA group. Green dots represent upregulated mRNAs, and blue dots represent downregulated mRNAs with statistical significance. **E**: A histogram of the number of genes up-/down-regulated in the two groups of DEGs. **F**: A Venn plot of DEGs shared by the IUA and normal groups and those shared by the Exo and IUA groups. **G**: Heat map of the top 100 DEGs shared by the IUA and normal groups and those of the Exo and IUA groups

To investigate the potential role of PMSC Exos in the modulation of IUA-induced endometrial injury, we subsequently performed whole transcriptome sequencing analysis on the normal, IUA, and Exo-treated groups. Through PCA (Figure 5a) and by using a sample correlation heatmap (Figure 5b), we observed clear differences and correlations among the groups. The volcano plot (Figure 5c,d) a total of 2039 upregulated DEGs and 2556 downregulated DEGs in the comparison between the IUA and normal groups at |log2fc| ≥1 and p <0.05 as the thresholds. In the comparison between the Exo and IUA groups, 2437 DEGs were upregulated, and 2316 DEGs were downregulated (Figure 5e). Detailed information on the DEGs is provided in Supplementary Tables 2 and 3. Afterward, a comprehensive analysis was conducted to examine the DEGs shared by the IUA and normal group and those shared by the Exos and IUA groups. As illustrated in Figure 5f, a total of 3980 DEGs were shared by these two groups. Additionally, a heatmap was used to visually represent the expression levels of the 100 most significant across the groups (Figure 5g).

### Functional enrichment analysis of the DEGs

First, we performed GO and KEGG enrichment analyses for the IUA versus normal groups and GSEA enrichment analysis for the Exo versus IUA group. Based on the findings presented in Supplementary Figure 1, the GO analysis of the IUA and normal group and the Exos and IUA groups primarily highlighted immune and inflammatory responses, and a negative correlation exists between the two datasets (Supplementary Figures 1a,d). Additionally, the KEGG results indicated that metabolic pathways were significantly enriched in the IUA versus normal groups and the Exo versus IUA groups (Supplementary Figures 1b,e). GSEA enrichment analysis validated the outcomes of the GO and KEGG enrichment analyses (Supplementary Figures 1c,f), and signaling pathways enriched in the head of the IUA versus normal group were enriched in the tail of the Exo versus IUA group.

**Fig. 6:**
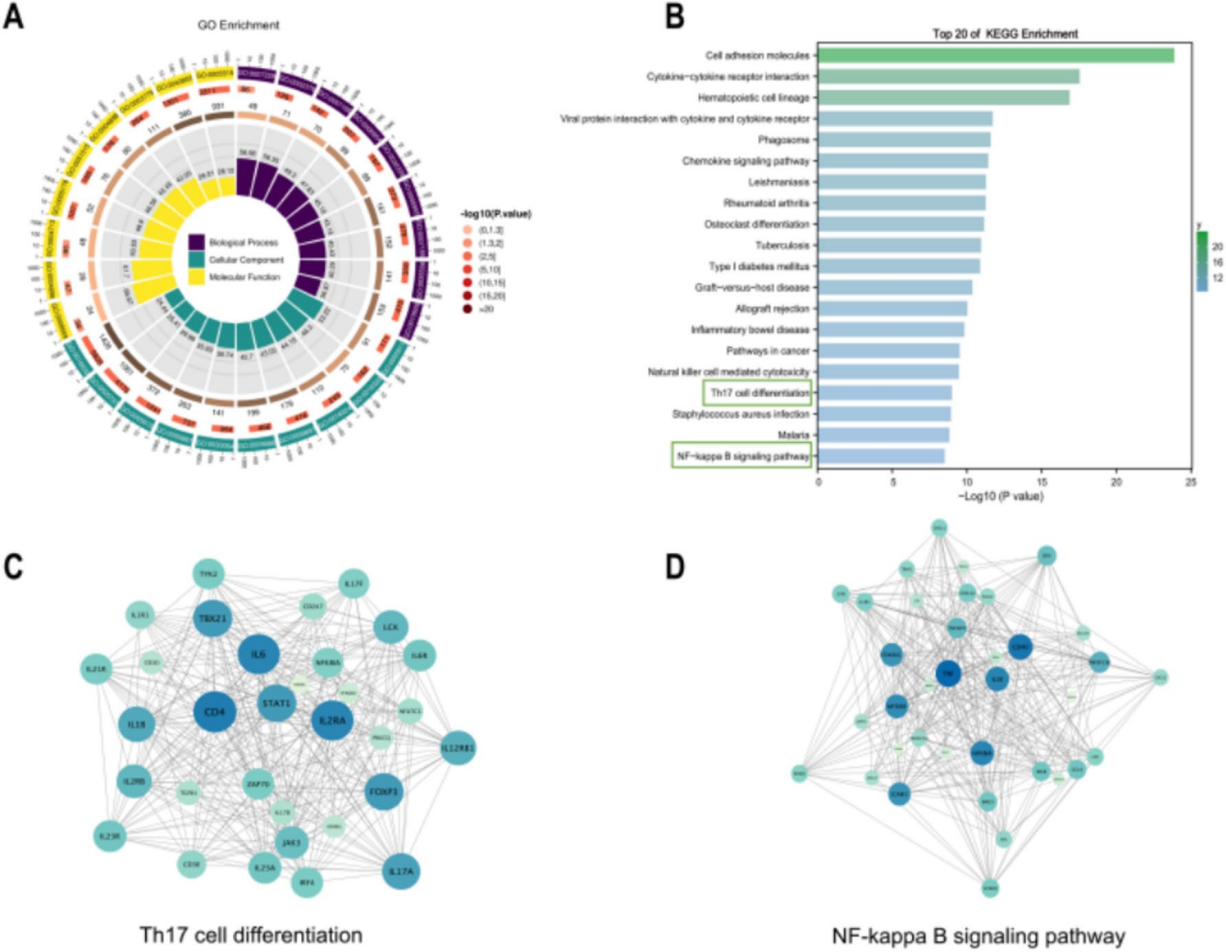
Functional enrichment analysis of DEGs **A**: GO enrichment analysis of DEGs. **B**: Top 20 KEGG enrichment analysis of DEGs. **C**: PPI network of Th17 cell differentiation-related proteins. **D**: PPI network of NF-kB signaling pathway–related proteins

Next, we conducted a comprehensive analysis of the GO and KEGG enrichment results for the DEGs shared by the IUA and normal groups and those shared by the Exo and IUA groups. As shown in Figure 6a, biological processes were primarily enriched in the integrin-mediated signaling pathway and immune system processes. The cellular components were mainly enriched in cell–cell junction and basolateral plasma membrane. In terms of molecular functions, we found significant enrichment in chemokine activity and cytokine receptor activity. As for KEGG analysis, Figure 6b depicts the top 20 enriched pathways, and a marked enrichment of cell adhesion molecules was observed.

NF-κB signaling and Th17/Treg balance may play important roles in MSC Exo treatment of IUA (Xue et al. 2015). To gain further insights into the protein interactions within these two pathways, we visualized the PPI network by using Cytoscape (Figures 6c,d). In the graph, node size reflects the degree of importance, and large nodes indicated significance. In the Th17/Treg balance, IL-6, CD4, and IL2RA act as hub genes, while in the NF-κB signaling pathway, TNF, MyD88, and CD40 act as hub genes.

### The miR-143 delivered by PMSC Exos targeted MyD88 to regulate the NF-κB signaling pathway

**Fig. 7:**
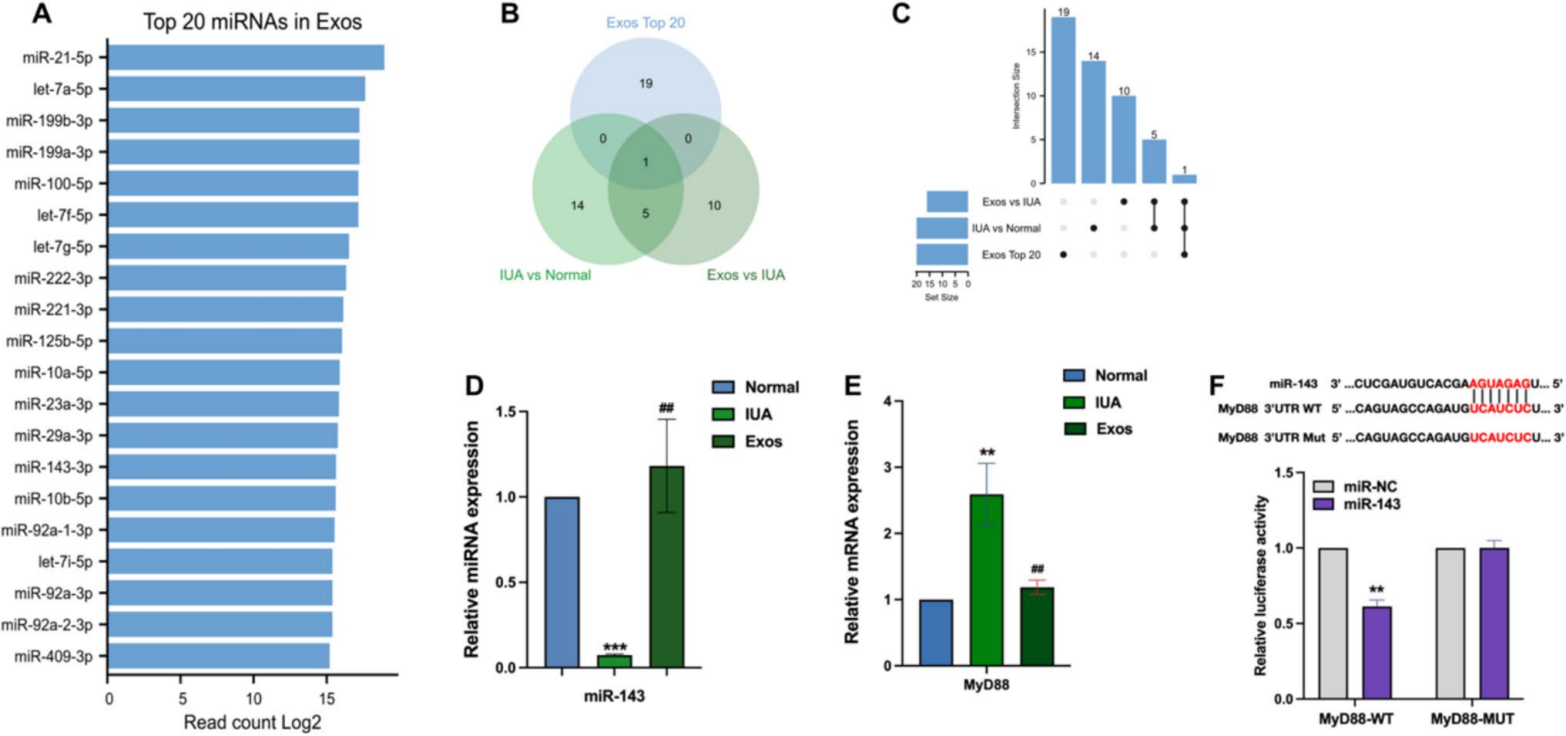
The miR-143 delivered by PMSC Exos targeted MyD88 **A**: Summary of the top 20 miRNAs in PMSC Exos. **B, C**: A Venn plot and a upset plot of shared by DEmiRNAs by the IUA vs normal groups, the Exos vs IUA groups and Top 20 miRNAs in the PMSC Exos. **D, E**: qRT-PCR analysis of miR-143-3p and MyD88 expressions in endometrial tissues (*compared with control group, #compared with IUA group). **F**: After transfection of miRNAs mimics and WT or MUT vectors into 293T, dual-luciferase reporter genes were performed

RNA is the most abundant Exos, and small RNA plays a major functional role (Fang et al. 2016). miRNA has received extensive attention due to its important role in regulating gene expression (Rutnam et al. 2013). To further explore the specific miRNAs carried by Exos, miRNA sequencing analysis was then performed on PMSC Exos, focusing on the 20 most abundant miRNAs present in PMSC Exos (Figure 7a). The intersection of the IUA vs normal groups , the Exos vs IUA groups, and the top 20 miRNAs in PMSC Exos were identified using Wayne and upset diagrams, and miR-143-3p was found to be handed over (Figures 7b,c). We speculated that Exos likely play a therapeutic role by delivering miR-143-3p into endometrial cells.

miRNAs primarily control post-transcriptional gene expression by inhibiting translation or promoting mRNA degradation in the cytoplasm. Therefore, we screened downstream genes targeted by miR-143 using the TargetScan and StarBase databases. The results showed that miR-143 may target MyD88. As previously demonstrated in the data analysis, MyD88 acts as a hub gene in the regulation of the NF-κB signaling pathway (Figures 6d). Thus, we hypothesize that the miR-143 delivered by PMSC Exos targets MyD88 to regulate the NF-κB signaling pathway.

First, we used RT-PCR to detect the expression levels of miR-143 and MyD88 after Exos treatment. The results showed that in the IUA disease model, miR-143 was significantly downregulated, while MyD88 was significantly upregulated. However, treatment with Exos significantly increased the expression of miR-143 and decreased the expression of MyD88 (Figures 7d,e). Subsequently, the dual-luciferase reporter assay results showed that miR-143 can bind to the 3'-UTR of MyD88, thereby inhibiting its expression (Figures 7f).

## Discussion

IUAs are caused by damage to the basal layer of the endometrium after repeated curettage, infection, and intrauterine surgery (Hooker et al. 2014). Many procedures include the transcervical resection of adhesion, hormone therapy, hyaluronic acid gels and transplantation of freeze-dried amnion grafts, which have been used to treat IUA and prevent recurrence (March 2011; Hooker et al. 2017; Roy et al. 2010; Gan et al. 2017). However, the treatment effect is not ideal, and endometrial regeneration and functional recovery remain major challenges. In this study, we demonstrated that exosomes derived from PMSC Exos significantly improved endometrial repair in a rat model of IUA. Through comprehensive transcriptomic and functional analyses, we identified that PMSC Exos deliver miR-143, which targets MyD88 to modulate the NF-κB signaling pathway, thereby reducing inflammation and fibrosis. These findings highlight the potential of PMSC Exos as a cell-free therapeutic strategy for IUA, offering a novel approach to address the limitations of current treatments.

MSCs have become the main cell sources for tissue regeneration due to their pluripotent differentiation, high expansion potential, and immunomodulatory ability. A variety of MSCs exert therapeutic effects on IUA (Zhang et al. 2018; Zheng et al. 2022; Min et al. 2021). However, stem cell therapy has some drawbacks, including the risk of immune rejection, need for surface stable cells, and potential risk of cancer or ectopic tissue development (Lin et al. 2021). Recent studies have shown that the therapeutic effect of stem cells is mainly driven by paracrine mechanism, and Exos have clear significance in the therapeutic effect of IUA (Liu and Wang 2022). Studies have shown that UCMSC Exos can promote endometrial regeneration and collagen remodeling, and restore fertility (Xin et al. 2020). In addition, Exo derived from BMSCs can promote endometrial repair in IUA animal models (Yao et al. 2019). In recent years, PMSCs have become a hot spot in MSC research because of their easy extraction, abundant sources, and few ethical issues (Patel et al. 2014). However, the therapeutic effect of secreted Exos on IUA remains to be explored. In this experiment, the rat IUA model constructed by electrocoagulation and the gross anatomical map of the rat uterus were observed, and endometrial damage was evaluated by HE and Masson staining, which confirmed the successful construction of the IUA animal model. Moreover, the endometrial thickness, number of glands, and fibrosis level of the damaged uterus after Exo treatment were measured, and the results showed that PMSC Exos significantly improved the damaged endometrium.

MSC Exos can deliver contained proteins, mRNA, miRNA, and other molecules to damaged cells, effectively inducing therapy-related signals to function (Fang et al. 2016). To explore the potential molecular mechanism of Exo therapy, we conducted transcriptome sequencing of mRNA and miRNA in the normal, IUA, and Exo groups and conducted differential expression analysis of the data to obtain 3980 common DEGs in the IUA and normal groups and in the Exo and IUA groups. KEGG/GO and GSEA were performed on DEGs. Result displayed that immune system processes, natural killer cell–mediated cytotoxicity, NF-κB signaling, Th17 cell differentiation, and other immunoinflammatory pathways and biological functions were enriched. Immune cells play an important role in the development of inflammatory response that triggers endometrial fibrosis. For example, Feng et al. found that the IUA group had increased proportions of endometrial CD45 cells, neutrophils, T-cells, and macrophages; decreased proportion of natural killer cells; and a high expression of LIGHT in CD4 T-cells and macrophages, compared with the control group (Abudukeyoumu et al. 2022). Duan et al. showed that the abnormal infiltration of inflammation-associated immune cells disrupts normal endometrial function in endometriosis (Duan et al. 2018). This result is consistent with our findings. Changes in endometrial immune cells in IUA are the key driving factors in finding the pathogenesis and therapeutic targets of IUA. MSC Exos can enhance the efficacy of IUA therapy by promoting the polarization of macrophages into M2 phenotype through TNF-α (Li et al. 2022). Another study showed that MSCs transplanted into the uterine cavity of mice can play an immunomodulatory role by secreting the anti-inflammatory cytokine IL-6 (Gan et al. 2017). These results confirm the abnormal distribution of immune cells and the importance of immune system regulation during endometrial injury and PMSC Exos, namely, NF-κB signaling and Th17 cell differentiation. Therefore, we constructed the PPI network maps of the two to explore the interactions among genes. Wang et al. proposed that NF-κB is a pathogenic factor of Ascherman syndrome, providing a novel idea for the prevention and treatment of IUA in patients (Wang et al. 2017). The activity of NF-κB signaling pathway in IUA endometrial tissues is significantly enhanced compared with that in normal endometrial tissues, and the NF-κB signaling may be involved in MSC transplantation for IUA treatment (Xue et al. 2015). RNA sequencing in another study showed that hUCB-MSCs regulate Th17/Treg balance and NF-κB signaling for IUA (Hua et al. 2022). The study was confirmed by PCR that MSC therapy can indeed reduce NF-κB-p28 protein levels. MSCs can regulate Th17 cell polarity into anti-inflammatory regulatory T-cells and reduce the ratio of Th17/Treg cells to perform immunomodulatory functions (Chen et al. 2020). The literature supports our findings. Exos play a therapeutic role primarily by delivering ncRNAs, and miRNA has received extensive interest due to its important role in the regulation of gene expression (Rutnam et al. 2013). Therefore, we speculated that miRNA may be involved in regulating Th17/Treg balance and NF-κB signaling pathway.

Next, we performed miRNA sequencing on Exos and conducted a comprehensive analysis in combination with the miRNA sequencing data from endometrial tissues. In PMSC-Exos, the content of miR-143-3p ranked relatively high. A Venn diagram was used to identify in the IUA vs normal groups, the Exos vs IUA groups and the top 20 miRNAs MSC-Exos. It was found that miR-143-3p is a common miRNA among these three groups. By using a database to screen for target genes of miR-143, we identified MyD88 as a target gene of miR-143. Notably, MyD88, as a hub gene in the NF-κB signaling pathway, plays an important role in the regulation of the pathway (Expression of Concern. 2021_)._ The dual-luciferase reporter assay confirmed that miR-143 can bind to the 3'UTR region of MyD88. The RT-qPCR experiment confirmed that in the IUA disease model, miR-143 was significantly downregulated, while MyD88 was significantly upregulated. However, after Exos treatment, the expression of miR-143 was significantly increased, and the expression of MyD88 was decreased. Moreover, MSC Exos are involved in the intercellular transfer of HAND2-AS1 and the treatment of rheumatoid arthritis through the miR-143-3p/NF-κB pathway (Su et al. 2021). Curcumin-treated MSC-derived Exos can regulate the NF-KB signaling pathway and play a therapeutic role by increasing the expression level of miR-143 in osteoarthritis (Qiu et al. 2020). This result further confirms our speculations. MSC Exos may be involved in the regulation of the NF-κB signaling pathway and can induce the polarization of M2 macrophages and Tregs to improve the inflammatory response of nephritis and other key organs (Sun et al. 2022). These results are consistent with our results, and thus we speculated that miR-143-3p carried by PMSC Exos can regulate the NF-κB signaling pathway and the balance of Th17/Treg cells by targeting MyD88, thereby participating in the regulation of the inflammatory environment in IUA, this mechanism still needs to be further explored in future experiments.

## Conclusions

In conclusion, our study demonstrates that PMSC Exos effectively restore endometrial function and morphology in a rat model of IUA. We identified key genes and signaling pathways involved in endometrial repair, highlighting the role of miR-143 in modulating the NF-κB signaling pathway and Th17/Treg cell balance through MyD88 targeting. These findings provide valuable insights into the therapeutic potential of PMSC Exos as a novel treatment strategy for IUA, offering a promising avenue for future clinical applications.

## Supplementary Information

Below is the link to the electronic supplementary material.

Supplementary file1 (XLSX 5198 KB)

Supplementary file2 (DOCX 272 KB)

## Data Availability

All sequencing data will be provided in supplementary materials after the article is approved.

## Abbreviations

IUA: Intrauterine adhesion
MSCs: Mesenchymal stem cells
BMSCs: Bone marrow stromal cells
UCMSCs: Umbilical cord mesenchymal stem cells
Exos: Exosomes
EMT: Epithelial–mesenchymal transition
AMSCs: Adipose-derived mesenchymal stem cells
PMSCs: Placental mesenchymal stem cells
PCA: Principal component analysis
DEGs: Differentially expressed genes
TEM: Transmission electron microscopy
NTA: Nanoparticle tracking analysis
HE: Hematoxylin–eosin
TRCA: Transcervical resection of adhesions
RT-PCR: Quantitative real-time reverse transcription polymerase chain reaction
DEmiRNAs: Differentially expressed miRNAs

## References

[CR1] Abudukeyoumu A et al (2022) A LIGHT-HVEM/LTbetaR axis contributes to the fibrosis of intrauterine adhesion. J Reprod Immunol 153:103693 35987137 10.1016/j.jri.2022.103693

[CR2] Chen Y et al (2017) Effects of aspirin and intrauterine balloon on endometrial repair and reproductive prognosis in patients with severe intrauterine adhesion: a prospective cohort study. Biomed Res Int 2017:8526104 28251159 10.1155/2017/8526104 PMC5303840

[CR3] Chen QH et al (2020) Mesenchymal stem cells regulate the Th17/Treg cell balance partly through hepatocyte growth factor in vitro. Stem Cell Res Ther 11(1):91 32111238 10.1186/s13287-020-01612-y PMC7049226

[CR4] Costa LA et al (2021) Functional heterogeneity of mesenchymal stem cells from natural niches to culture conditions: implications for further clinical uses. Cell Mol Life Sci 78(2):447–467 32699947 10.1007/s00018-020-03600-0 PMC7375036

[CR5] Duan J et al (2018) The M2a macrophage subset may be critically involved in the fibrogenesis of endometriosis in mice. Reprod Biomed Online 37(3):254–268 30314882 10.1016/j.rbmo.2018.05.017

[CR6] Expression of Concern: miR-143-3p impacts on pulmonary inflammatory factors and cell apoptosis in mice with mycoplasmal pneumonia by regulating TLR4/MyD88/NF-kappaB pathway*.* Biosci Rep, 2021. **41**(4): BSR-20193419_EOC 10.1042/BSR-20193419_EOC PMC8015334 33786576

[CR7] Fang S et al (2016) Umbilical cord-derived mesenchymal stem cell-derived exosomal microRNAs suppress Myofibroblast differentiation by inhibiting the transforming growth factor-beta/SMAD2 pathway during wound healing. Stem Cells Transl Med 5(10):1425–1439 27388239 10.5966/sctm.2015-0367 PMC5031180

[CR8] Gan L et al (2017) Human amniotic mesenchymal stromal cell transplantation improves endometrial regeneration in rodent models of intrauterine adhesions. Cytotherapy 19(5):603–616 28285950 10.1016/j.jcyt.2017.02.003

[CR9] Gan L et al (2017) Efficacy of freeze-dried amnion graft following hysteroscopic adhesiolysis of severe intrauterine adhesions. Int J Gynaecol Obstet 137(2):116–122 28170094 10.1002/ijgo.12112

[CR10] Gao M et al (2022) Mesenchymal stem cells therapy: a promising method for the treatment of uterine scars and premature ovarian failure. Tissue Cell 74:101676 34798583 10.1016/j.tice.2021.101676

[CR11] Hooker AB et al (2014) Systematic review and meta-analysis of intrauterine adhesions after miscarriage: prevalence, risk factors and long-term reproductive outcome. Hum Reprod Update 20(2):262–78 24082042 10.1093/humupd/dmt045

[CR12] Hooker AB et al (2017) Prevalence of intrauterine adhesions after the application of hyaluronic acid gel after dilatation and curettage in women with at least one previous curettage: short-term outcomes of a multicenter, prospective randomized controlled trial. Fertil Steril 107(5):1223-1231e3 28390688 10.1016/j.fertnstert.2017.02.113

[CR13] Hua Q et al (2022) Human umbilical cord blood-derived MSCs trans-differentiate into endometrial cells and regulate Th17/Treg balance through NF-kappaB signaling in rabbit intrauterine adhesions endometrium. Stem Cell Res Ther 13(1):301 35841027 10.1186/s13287-022-02990-1 PMC9284747

[CR14] Kelleher AM et al (2018) Uterine glands coordinate on-time embryo implantation and impact endometrial decidualization for pregnancy success. Nat Commun 9(1):2435 29934619 10.1038/s41467-018-04848-8 PMC6015089

[CR15] Koskas M et al (2010) Office hysteroscopy for infertility: a series of 557 consecutive cases. Obstet Gynecol Int 2010:168096 20396413 10.1155/2010/168096 PMC2855076

[CR16] Lee BC, Kang I, Yu KR (2021) Therapeutic features and updated clinical trials of mesenchymal stem cell (MSC)-derived exosomes. J Clin Med 10(4):711 33670202 10.3390/jcm10040711 PMC7916919

[CR17] Leung RK, Lin Y, Liu Y (2021) Recent advances in understandings towards pathogenesis and treatment for intrauterine adhesion and disruptive insights from single-cell analysis. Reprod Sci 28(7):1812–1826 33125685 10.1007/s43032-020-00343-y PMC8189970

[CR18] Li J et al (2022) Tumor necrosis factor-alpha-primed mesenchymal stem cell-derived exosomes promote M2 macrophage polarization via Galectin-1 and modify intrauterine adhesion on a novel murine model. Front Immunol 13:945234 36591221 10.3389/fimmu.2022.945234 PMC9800892

[CR19] Li X et al (2023) Umbilical cord mesenchymal stem cell-derived exosomes reverse endometrial fibrosis by the miR-145-5p/ZEB2 axis in intrauterine adhesions. Reprod Biomed Online 46(2):234–243 36567149 10.1016/j.rbmo.2022.05.018

[CR20] Lin J et al (2021) Microenvironment-protected exosome-hydrogel for facilitating endometrial regeneration, fertility restoration, and live birth of offspring. Small 17(11):e2007235 33590681 10.1002/smll.202007235

[CR21] Liu HD, Wang SW (2022) Role of noncoding RNA in the pathophysiology and treatment of intrauterine adhesion. Front Genet 13:948628 36386826 10.3389/fgene.2022.948628 PMC9650223

[CR22] Liu F et al (2019) Hyaluronic acid hydrogel integrated with mesenchymal stem cell-secretome to treat endometrial injury in a rat model of asherman’s syndrome. Adv Healthc Mater 8(14):e1900411 31148407 10.1002/adhm.201900411 PMC7045702

[CR23] Liu Y et al (2020) Collagen scaffold with human umbilical cord mesenchymal stem cells remarkably improves intrauterine adhesions in a rat model. Gynecol Obstet Invest 85(3):267–276 32289792 10.1159/000505691

[CR24] Liu NN et al (2022) Mycobiome dysbiosis in women with intrauterine adhesions. Microbiol Spectr 10(4):e0132422 35730962 10.1128/spectrum.01324-22 PMC9431258

[CR25] Ma H et al (2020) Intrauterine transplantation of autologous menstrual blood stem cells increases endometrial thickness and pregnancy potential in patients with refractory intrauterine adhesion. J Obstet Gynaecol Res 46(11):2347–2355 32856391 10.1111/jog.14449

[CR26] Mao Y et al (2023) Human amniotic mesenchymal stem cells promote endometrium regeneration in a rat model of intrauterine adhesion. Cell Biol Int 47(1):75–85 36317446 10.1002/cbin.11951

[CR27] Maraldi T, Russo V (2022) Amniotic fluid and placental membranes as sources of stem cells: progress and challenges. Int J Mol Sci 23(10):5362 35628186 10.3390/ijms23105362 PMC9141978

[CR28] March CM (2011) Management of asherman’s syndrome. Reprod Biomed Online 23(1):63–76 21549641 10.1016/j.rbmo.2010.11.018

[CR29] Min J et al (2021) Phenotype and biological characteristics of endometrial mesenchymal stem/stromal cells: a comparison between intrauterine adhesion patients and healthy women. Am J Reprod Immunol 85(6):e13379 33206449 10.1111/aji.13379

[CR30] Patel J et al (2014) Novel isolation strategy to deliver pure fetal-origin and maternal-origin mesenchymal stem cell (MSC) populations from human term placenta. Placenta 35(11):969–71 25239220 10.1016/j.placenta.2014.09.001

[CR31] Qiu G et al (2019) Functional proteins of mesenchymal stem cell-derived extracellular vesicles. Stem Cell Res Ther 10(1):359 31779700 10.1186/s13287-019-1484-6 PMC6883709

[CR32] Qiu B et al (2020) Curcumin reinforces MSC-derived exosomes in attenuating osteoarthritis via modulating the miR-124/NF-kB and miR-143/ROCK1/TLR9 signalling pathways. J Cell Mol Med 24(18):10855–10865 32776418 10.1111/jcmm.15714 PMC7521270

[CR33] Roy KK et al (2010) Reproductive outcome following hysteroscopic adhesiolysis in patients with infertility due to asherman’s syndrome. Arch Gynecol Obstet 281(2):355–61 19455349 10.1007/s00404-009-1117-x

[CR34] Rutnam ZJ, Wight TN, Yang BB (2013) miRNAs regulate expression and function of extracellular matrix molecules. Matrix Biol 32(2):74–85 23159731 10.1016/j.matbio.2012.11.003 PMC4106267

[CR35] Su Y et al (2021) Mesenchymal stem cell-originated exosomal lncRNA HAND2-AS1 impairs rheumatoid arthritis fibroblast-like synoviocyte activation through miR-143-3p/TNFAIP3/NF-kappaB pathway. J Orthop Surg Res 16(1):116 33549125 10.1186/s13018-021-02248-1 PMC7866436

[CR36] Sun W et al (2022) Mesenchymal stem cells-derived exosomes ameliorate lupus by inducing M2 macrophage polarization and regulatory T cell expansion in MRL/lpr mice. Immunol Invest 51(6):1785–1803 35332841 10.1080/08820139.2022.2055478

[CR37] Wang X et al (2017) Elevated NF-kappaB signaling in asherman syndrome patients and animal models. Oncotarget 8(9):15399–15406 28148903 10.18632/oncotarget.14853 PMC5362494

[CR38] Xin L et al (2020) A scaffold laden with mesenchymal stem cell-derived exosomes for promoting endometrium regeneration and fertility restoration through macrophage immunomodulation. Acta Biomater 113:252–266 32574858 10.1016/j.actbio.2020.06.029

[CR39] Xue X et al (2015) The overexpression of TGF-beta and CCN2 in Intrauterine adhesions involves the NF-kappaB signaling pathway. PLoS One 10(12):e0146159 26719893 10.1371/journal.pone.0146159 PMC4697802

[CR40] Yao Y et al (2019) Exosomes derived from mesenchymal stem cells reverse EMT via TGF-beta1/Smad pathway and promote repair of damaged endometrium. Stem Cell Res Ther 10(1):225 31358049 10.1186/s13287-019-1332-8 PMC6664513

[CR41] Zhang L et al (2018) Therapeutic effect of human umbilical cord-derived mesenchymal stem cells on injured rat endometrium during its chronic phase. Stem Cell Res Ther 9(1):36 29433563 10.1186/s13287-018-0777-5 PMC5810045

[CR42] Zhao S et al (2020) Exosomes derived from adipose mesenchymal stem cells restore functional endometrium in a rat model of intrauterine adhesions. Reprod Sci 27(6):1266–1275 31933162 10.1007/s43032-019-00112-6

[CR43] Zheng Y et al (2022) circPTP4A2-miR-330-5p-PDK2 signaling facilitates in vivo survival of HuMSCs on SF-SIS scaffolds and improves the repair of damaged endometrium. Oxid Med Cell Longev 2022:2818433 35571241 10.1155/2022/2818433 PMC9106474

