# Supplementary figures and images for: Placental mesenchymal stem cell–derived exosomes treat endometrial injury in a rat model of intrauterine adhesions
Source: Mol Genet Genomics. 2025 Mar 25;300(1):36. doi: 10.1007/s00438-025-02241-x (PMC11933197; doi:10.1007/s00438-025-02241-x)

Supplementary Figures

Supplementary figure1


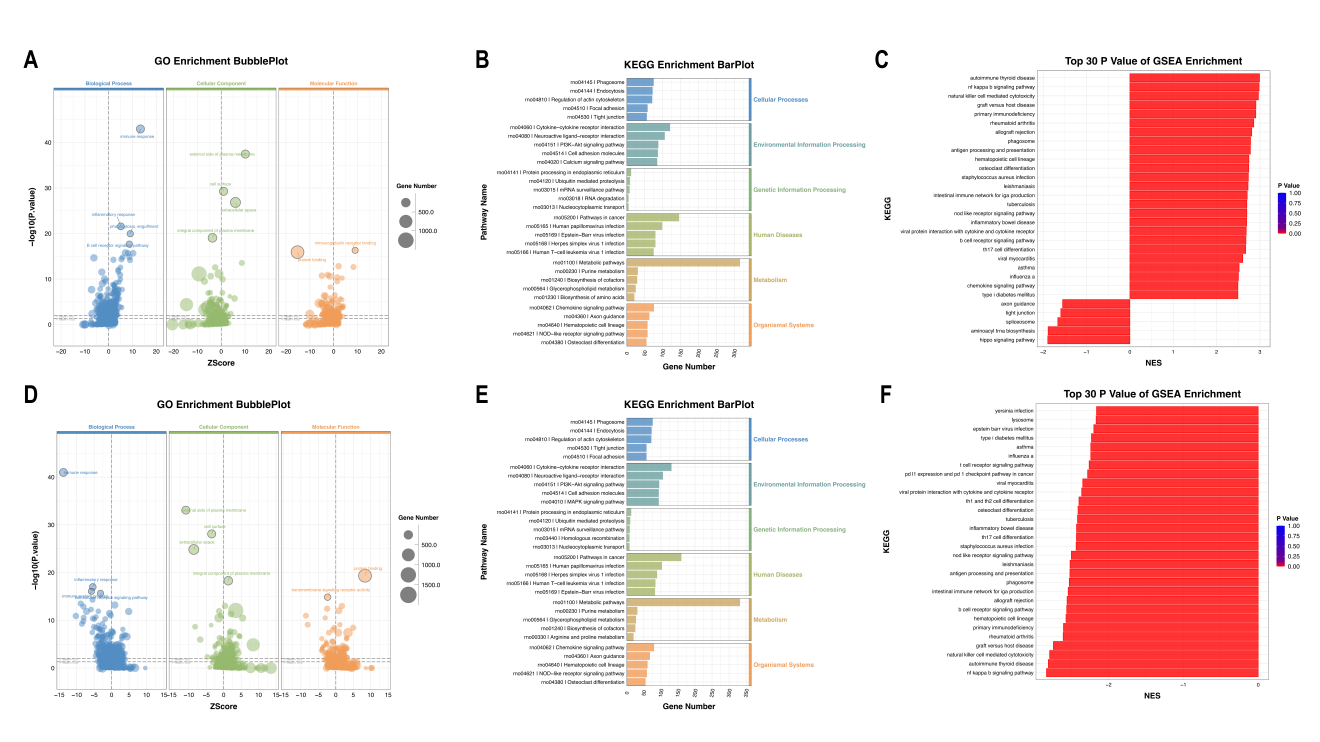

Supplement: Supplementary file 2 — Supplementary file2 (DOCX 272 KB) [file 438_2025_2241_MOESM2_ESM.docx]
